# Paediatric Erdheim-Chester Disease in the Lateral Ventricle: A Case Report and Review of the Literature

**DOI:** 10.3389/fonc.2022.835076

**Published:** 2022-04-14

**Authors:** Yimei Ma, Xia Guo, Zhi Wan, Hanmin Liu, Ju Gao

**Affiliations:** ^1^Department of Pediatrics, West China Second University Hospital, Sichuan University, Chengdu, China; ^2^Key Laboratory of Birth Defects and Related Diseases of Women and Children (Sichuan University), Ministry of Education, Chengdu, China; ^3^Department of Pediatric Pulmonology and Immunology, West China Second University Hospital, Sichuan University, Chengdu, China; ^4^Key Laboratory of Chronobiology (Sichuan University), National Health Commission of China, Chengdu, China; ^5^The Joint Laboratory for Lung Development and Related Diseases of West China Second University Hospital, Sichuan University and School of Life Sciences of Fudan University, West China Institute of Women and Children’s Health, West China Second University Hospital, Sichuan University, Chengdu, China; ^6^Sichuan Birth Defects Clinical Research Center, West China Second University Hospital, Sichuan University, Chengdu, China

**Keywords:** Erdheim Chester disease, lateral ventricle, CD68, CD1a, BRAF*^V600E^*

## Abstract

Erdheim-Chester disease (ECD) is a rare non-Langerhans cell histiocytosis caused by the expression of CD68-positive and CD1a-negative foam tissue cells, which is polar in pediatric patients. The study reports a case of an 8-year-old Chinese boy who presented with polydipsia and polyuria for 4 years, followed by central nervous system symptoms. Magnetic resonance imaging (MRI) showed a large lesion in the lateral ventricle. The histiocytes stained positively for CD68, CD163 and negatively for CD1a, glial fibrillary acidic protein (GFAP) and langerin, and were partially positive for S100 by immunohistochemical assay. More importantly, BRAFV*^600E^* staining was positive in tissue, and the BRAF*^V600E^* mutations was also detected by real-time quantitative PCR (RT-qPCR) in the intracranial lesion tissue. According to our review of the literature, this is a rare case of ECD in the ventricle, with a younger age.

## Introduction

Erdheim-Chester disease (ECD) is a rare non-Langerhans cell histocytosis (LCH) (1), originally described by Jakob Erdheim and William Chester as “Lipid Granulomatosis” in 1930 (2). Although the number of patients with ECD has increased over the past few decades (3, 4), little is known about the pathogenesis of the disease. At the same time, ECD can present with complex manifestations of multisystem involvement (3, 5, 6), including cerebellar signs, urinary collapse, hypopituitarism, lung and heart disease, and its incidence is rare and may inevitably lead to misdiagnosis. In this study, a unique boy who reported the initial symptoms of polydipsia, polyuria and limited left eye movement was ultimately identified as having ECD during ventricular initiation. This instructive case highlights the unusual pattern of ECD in children, which may potentially affect the diagnostic mechanisms of the central nervous system (CNS) and should be considered for further investigation for accurate early diagnosis and effective treatment.

## Case Presentation

**Figure 1:**
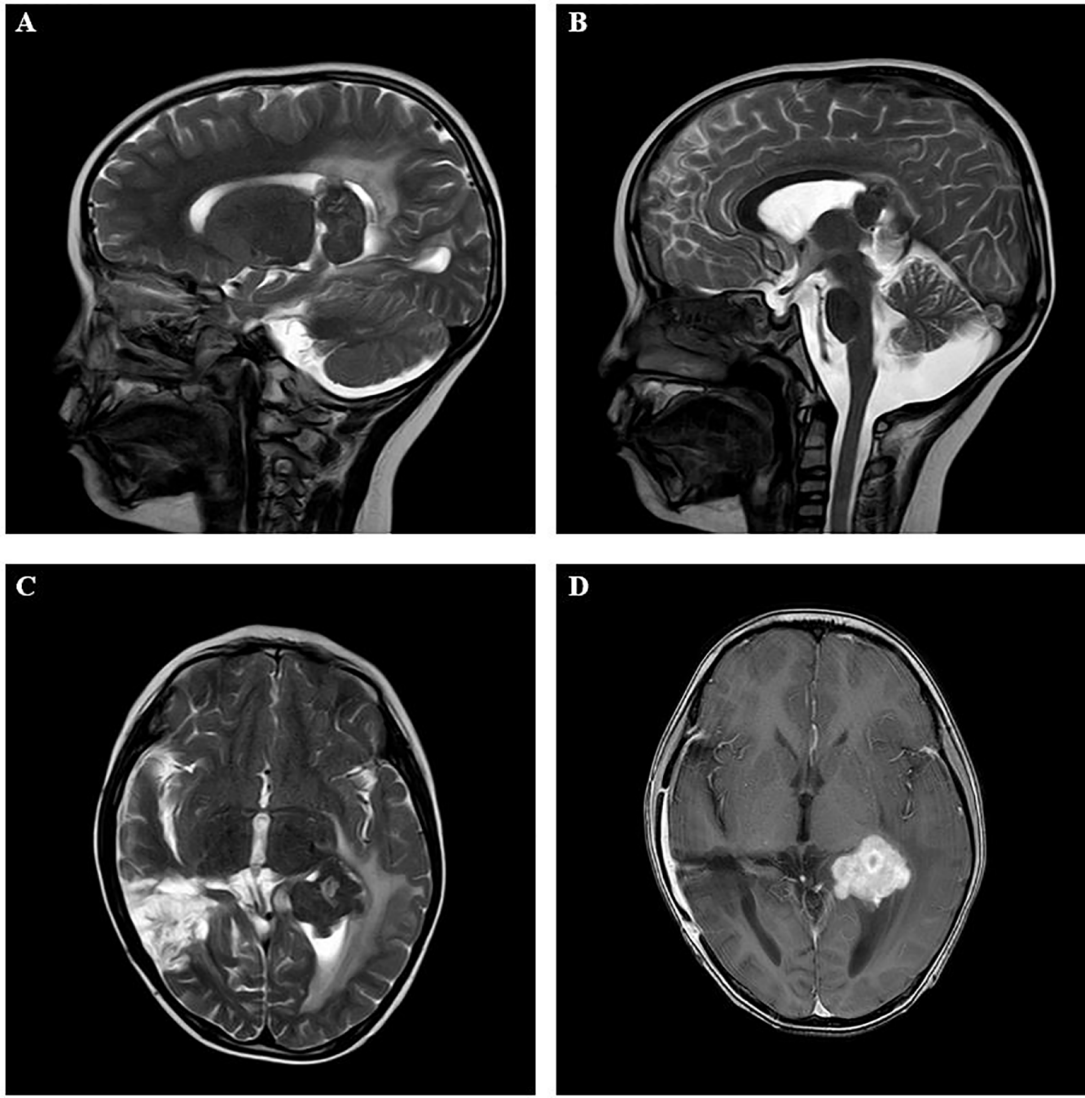
Imaging results in the intracranial lesion tissue. The results of MRI showed the lateral ventricle expansion, with abnormal signal mass shadow (size about 3.3×3.1×3.5cm) in the lateral ventricle. (**A**, **B**: sagittal position; **C**, **D**: horizontal position). MRI, Magnetic resonance imaging.

**Figure 2:**
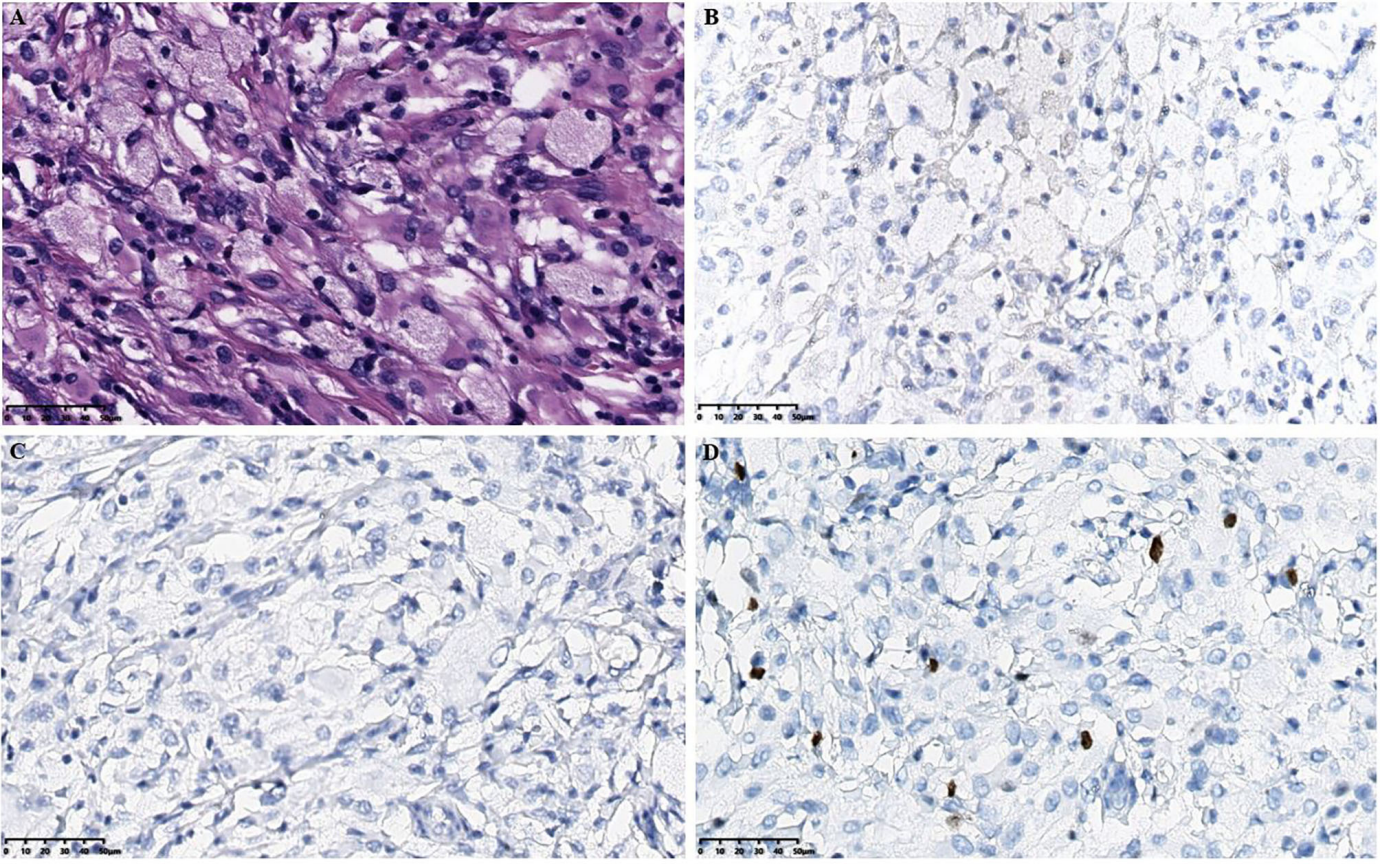
Histopathological results in the intracranial lesion tissue. **(A)** Hematoxylin-eosin staining in the intracranial lesion tissue showed abundant cytoplasm in the ventricular soft tissue mass cells with foamy or eosinophilic histiocytic infiltration (scale bar is 50μm). **(B)** Immunohistochemical CD1a staining showed negative tissue cells (scale bar is 50μm). **(C)** Immunohistochemical langerin staining showed negative tissue cells (scale bar is 50μm). **(D)** Immunohistochemical Ki-67 staining showed a small amount of positive tissue cells (scale bar is 50μm).

An 8-year-old Chinese boy presented with “polydipsia and polyuria for 4 years and left eye movement limitation”. Within 4 years, the patient repeatedly developed polydipsia and polyuria, but there was no change in body weight, fever, diarrhea, bloody stool, convulsions or other discomfort, and the child was not treated. Recently, children showed limited left eye movement, fixation on the nasal side, limited outreach, visual shadows, no vision loss or vision loss, and no headache, dizziness, fever, vomiting, or depression. Then, the child went to the hospital for magnetic resonance imaging (MRI) examination, which showed an intracranial mass in the lateral ventricles **(** **Figure 1****).** Physical examination showed normal muscle strength of the limbs and no headache, fever, vomiting, drowsiness or poor appetite. Subsequently, the patient underwent massive mass resection in the lateral ventricle. Macroscopic observation showed that the lesion was located in the lateral ventricle, which was solid, tough and yellow, with abundant blood supply and extensive adhesion in the lateral ventricle. The patient did not receive chemotherapy after surgery but had normal eye movements and no clinical symptoms, such as vomiting, headache and convulsions. However, the child suddenly developed an impairment of consciousness, accompanied by neurological symptoms of jet vomiting, headache, and nonsense one month after the surgery. Laboratory examination: white blood cell (WBC) 4.6 × 10^9^/L, hemoglobin (Hb) 139 g/L, platelet (PLT) 354 × 10^9^/L, absolute neutrophil count (ANC) 2.86×10^9^/L, C-reactive protein (CRP) 5.7 mg/L; Vitamin D determination 10.80 ng/ml; In addition, liver and kidney function, electrolyte, methyl work, disseminated intravascular coagulation, hemolysis, cell immunity, humoral immunity, iron deficiency full set are normal, as well as the results of the cardiac ultrasound, urinary tract ultrasound, chest computed tomography (CT) scan are also normal. The results of the second head CT showed abnormal density shadows in both cerebral hemispheres, with solid components mainly located in the ventricle, suggesting a possibility of mass and irregular large patchy low density in the paraventricular parenchyma. However, the results of single photon emission computed tomography (SPECT) showed no signs of tumor metastasis. In addition, the hematoxylin-eosin (H&E) staining in the intracranial lesion tissue showed abundant cytoplasm in the ventricular soft tissue mass cells with foamy or eosinophilic histiocytic infiltration, and the histiocytes stained negatively for CD1a and langerin, and also found a Ki67-positive rate of approximately 5% (**Figure 2**).

**Figure 3:**
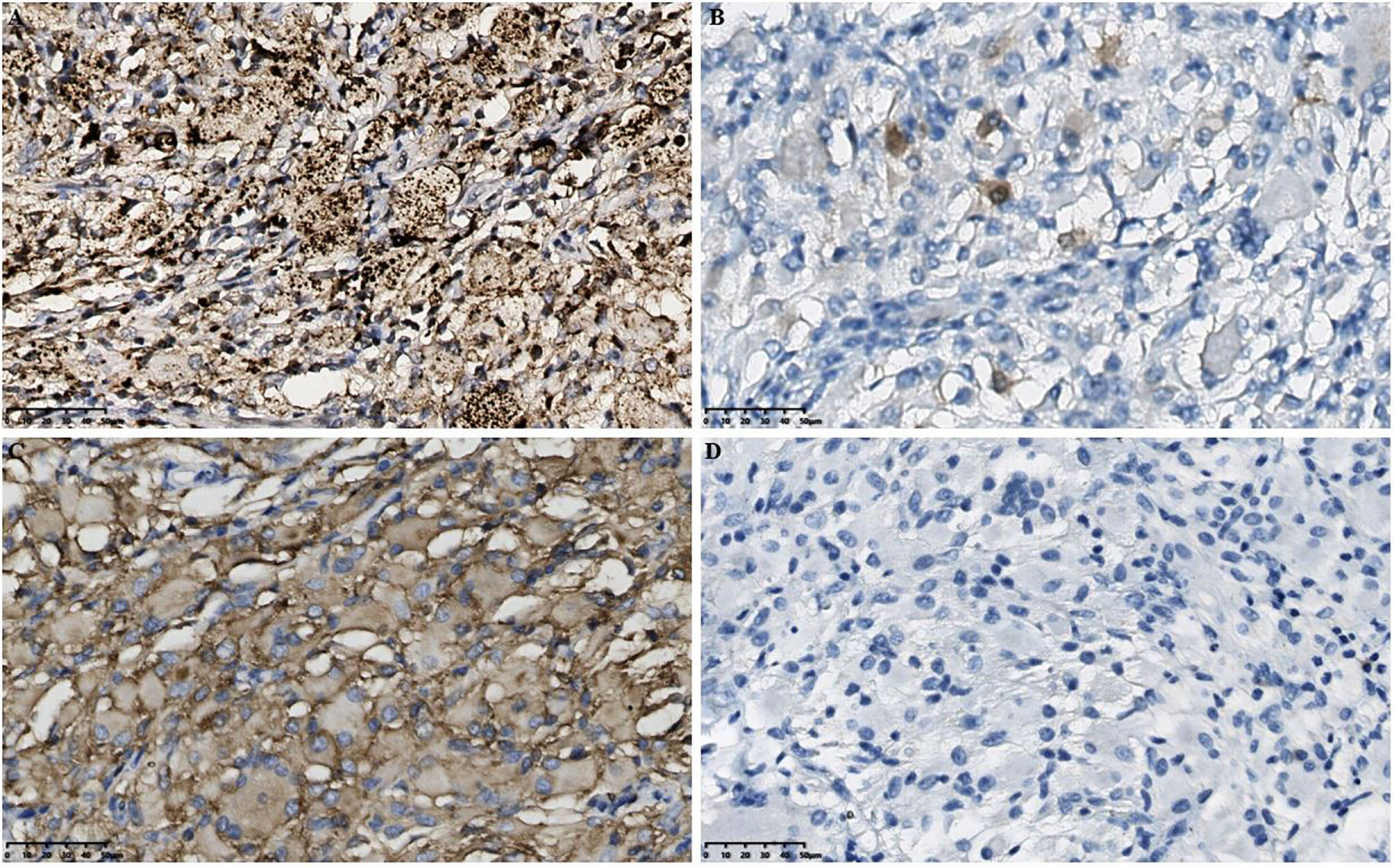
Histopathological results in the intracranial lesion tissue. **(A)** Immunohistochemical CD68 staining showed positive tissue cells (scale bar is 50μm). **(B)** Immunohistochemical S-100 staining showed weakly positive tissue cells (scale bar is 50μm). **(C)** Immunohistochemical CD163 staining showed positive tissue cells (scale bar is 50μm). **(D)** Immunohistochemical GFAP staining showed negative tissue cells (scale bar is 50μm). GFAP, Glial fibrillary acidic protein.

**Figure 4:**
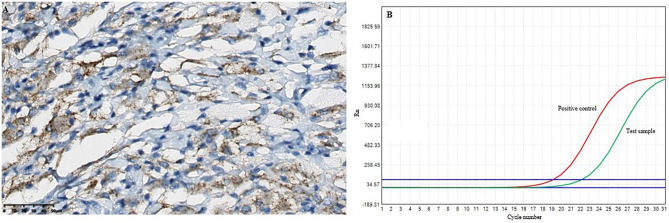
The results of the BRAF*^V600E^* mutations in the intracranial lesion tissue. **(A)** Immunohistochemical BRAF*^V600E^* staining showed positive tissue cells (scale bar is 200/50μm). **(B)** and the BRAF*^V600E^* mutations was detected by RT-qPCR.

The histiocytes stained positively for CD68, CD163, and negatively for glial fibrillary acidic protein (GFAP) and were partially positive for S-100 by immunohistochemical assay (**Figure 3**). Based on the above results, we initially suspect that it may be an ECD. Therefore, to confirm this hypothesis, we further tested for the presence of BRAF*^V600E^* mutation in diseased tissue, then the pathological results confirmed positive BRAF*^V600E^* staining, and the BRAF*^V600E^* mutations was also detected by real-time quantitative PCR (RT-qPCR) in the intracranial lesion tissue (**Figure 4**). Subsequently, the patient was treated with dexamethasone combined with a vincristine regimen, followed by an oral V600E-targeted drug (darafenib). The symptoms of the patient improved, and outpatient follow-up was performed. The neurological symptoms were improved in pediatric ECD patients, as well as normal follow-up through outpatient visits.

## Discussion

The diagnosis of ECD relies mainly on clinical, laboratory and imaging examinations (7, 8). Additionally, major immunohistochemistry shows that CD68 is positive and CD1a is negative (9). To our knowledge, previous literature has confirmed that a few cases of pediatric ECD have been reported. Therefore, the correct diagnosis of ECD remains a challenge, as systemic pediatric histiocytic disease is commonly diagnosed as pediatric LCHs. Notably, previous studies have confirmed that ECD mainly affects adults, with a slight predominance in males. For example, Boyd et al. (10) reported 62 patients (47 males and 15 females) with ECD, with a mean age of 54 years (range 22-74 years), a mean age of onset of 46 years (range 16-74 years), and a mean time of diagnosis of 4.2 years (range 0-24 years).

Previous studies have demonstrated that ECD can manifest as multisystemic involvement, including long bone involvement as the most common manifestation of ECD, accounting for 80% of patients (11). In addition, the CNS symptoms in ECD exhibit a variety, with cerebellar and pyramidal syndrome being the most common neurological signs, and other features described include seizures, headache, neuropsychiatric signs, cognitive impairment, sensory impairment, and cranial nerve palsy (10, 12). Multisystemic organs, including diabetes insipidus (13), skin disease with xanthelasma-like lesions (XLL) (14), perirenal fat infiltration (15), and lung and heart involvement (16), are all affected, which provides a challenge for pediatricians to identify differences from ECD. In this study, the case was complex and rare because the patient first developed clinical manifestations of polydipsia and polyuria, followed by limitation of movement in the left eye. After the MRI examination, a large space occupation was found in the lateral ventricle, and the brain tissue pathology biopsy showed foam cell infiltration. The histiocytes stained positively for CD68 and negatively for CD1a and langerin and were partially positive for S-100 by immunohistochemical assay. BRAF*^V600E^* mutation was also detected. Based on the above results, the child was diagnosed with ECD. Since it is very rare in childhood, it is difficult for pediatricians to clinically distinguish ECD from LCH and Rosai-Dorfman disease (RDD) with similar imaging patterns. Therefore, the histopathological features help confirm the diagnosis, and specific positive immunohistochemical markers are usually detected in the diseased tissue for ECD, RDD and LCH.

As the molecular pathogenesis of ECD disease has been explored, many therapeutic options, including interferon-α (IFN-α), BRAF, MEK targeted therapy, and anti-cytokine biotherapy, have been widely used to treat ECD. IFN-α has been shown to regulate dendritic cell maturation and activation (15) and is often recommended as a first-line treatment for nonlife-threatening presentations, which has been shown to improve survival. In recent years, almost 54%-57.5% of patients with ECD have been reported to carry BRAF*^V600E^*-activating mutations in the RAS-RAF-MEK-extracellular signal-regulated kinase signaling pathway (1, 17). Cohen et al (18) investigated the efficacy of a BRAF inhibitor (vemurafenib) and MEK inhibitor (cobimetinib) in 54 patients with ECD in 2017 and found that the median time for relapse was 6 months in 75% of patients with discontinuation of treatment. However, 10 patients subsequently resumed BRAF inhibitor treatment, resulting in improvement in all patients. Similarly, Diamond et al (19) showed that cobimetinib is effective in ECD patients with an overall remission rate of 89% and has not acquired resistance to date. There is no doubt that our understanding of the pathogenesis of ECD will continue to improve and may evolve into new treatments. In addition, biotherapeutic strategies targeting inflammatory cytokines have different clinical efficacies for ECD. For example, infliximab (targeting TNF-α) (20) and anakinra (targeting IL-1β) (21) are the most commonly used biotherapeutic therapies, and tocilizumab is also a biotherapeutic therapy targeting the IL-6 receptor (22). However, biotherapies targeting cytokines are still currently limited, and their clinical efficacy needs to be further explored in the future, especially in patients with severe inflammatory symptoms.

According to our review of the literature, this is a case of early polydipsia and polyuria as the main symptom, followed by neurological onset, with a younger age. Compared to previously reported cases, the current case is unique in that it occurs in the lateral ventricle. In conclusion, the study will make it likely easier for pediatricians and neurosurgeons to identify manifestations of ECD and correlate them with histopathological features.

## Data Availability Statement

The original contributions presented in the study are included in the article/supplementary materials. Further inquiries can be directed to the corresponding authors.

## Ethics Statement

The studies involving human participants were reviewed and approved by West China Second University Hospital. Written informed consent to participate in this study was provided by the participants’ legal guardian/next of kin. Written informed consent was obtained from the individual(s), and minor(s)’ legal guardian/next of kin, for the publication of any potentially identifiable images or data included in this article.

## Author Contributions

YM performed the histological images analysis. XG reviewed and edited the revised paper. ZW performed the radiological images analysis. YMM, XG, ZW, JG, HL performed the manuscript preparation. All authors contributed to the article and approved the submitted version.

## Funding

The study was supported by the Science and Technology Department of Sichuan Province (No.2022YFS0061), and it was supported by the West China Second University Hospital of Sichuan University (No.KX144).

## Conflict of Interest

The authors declare that the research was conducted in the absence of any commercial or financial relationships that could be construed as a potential conflict of interest.

## Publisher’s Note

All claims expressed in this article are solely those of the authors and do not necessarily represent those of their affiliated organizations, or those of the publisher, the editors and the reviewers. Any product that may be evaluated in this article, or claim that may be made by its manufacturer, is not guaranteed or endorsed by the publisher.
